# Información de la Enfermedad de Alzheimer para Latinos: A Study Framework for Alzheimer’s Genetic Risk Disclosure in an Urban, Latino Population

**DOI:** 10.1002/alz.090102

**Published:** 2025-01-09

**Authors:** Sophia Rodriguez, Daniela Diaz Caro, John Brandon Wetmore, Jonathan D Godinez, Itzel A Camarillo, Jill S Goldman, Wendy R. Uhlmann, Rebecca Ferber, Drew Blasco, Maria Caban, Cheng‐Shiun Leu, Ana F Abraido‐Lanza, Rafael A. Lantigua, Wendy K Chung, J. Scott Roberts, Karolynn Siegel, Ruth Ottman

**Affiliations:** ^1^ Columbia University Irving Medical Center, New York, NY USA; ^2^ Columbia University, New York, NY USA; ^3^ University of Michigan Medical School, Ann Arbor, MI USA; ^4^ University of Michigan, Ann Arbor, MI USA; ^5^ University of Nevada, Las Vegas, Las Vegas, NV USA; ^6^ Harvard Medical School, Boston, MA USA; ^7^ Boston Children’s Hospital, Boston, MA USA; ^8^ University of Michigan School of Public Health, Ann Arbor, MI USA; ^9^ New York State Psychiatric Institute, New York, NY USA

## Abstract

**Background:**

The IDEAL study is a randomized clinical trial investigating the psychosocial, behavioral, and cognitive impacts of genetic risk disclosure for late‐onset Alzheimer’s disease (LOAD) among Latinos.

**Methods:**

We used address‐based sampling in northern Manhattan to recruit Latinos aged 40‐64 for a community‐based survey and clinical trial. Data collection encompasses demographics, Alzheimer’s disease (AD) family history, knowledge and beliefs about AD and genetics, current mental health status, acculturation, impact of COVID‐19, familism, fatalism, caregiver status, and prior AD genetic testing. Eligible participants are invited to complete an education session and provide informed consent before receiving genetic testing for *APOE*, a key LOAD predictor. The clinical trial component will include 400 participants, who are randomized to learn their LOAD risk by age 85 based on either Latino ethnicity and family history alone or the same factors plus *APOE* genotype. Risk information is provided through semi‐structured genetic counseling sessions. Psychological impacts, health‐related behavioral changes, and memory performance are evaluated six weeks, nine months, and fifteen months post‐dislosure via surveys and in‐depth qualitative interviews.

**Results:**

From 91,433 invited households, 5,542 (6.1%) responded, and 2,087 (2.3%) were eligible and completed the Baseline survey. Most were 40‐49 years old (41.2%), women (70.5%), identified as Dominican (53.7%), had some college education (63.2%), and completed the survey in English (74.6%). About half reported a family history of AD (48.6%).

**Conclusion:**

This study addresses gaps in understanding of impacts of AD genetic risk disclosure among Latinos and contributes valuable insights through its mixed‐methods approach.

